# Chronological Age Assessment in Young Individuals Using Bone Age Assessment Staging and Nonradiological Aspects: Machine Learning Multifactorial Approach

**DOI:** 10.2196/18846

**Published:** 2020-09-21

**Authors:** Ana Luiza Dallora, Ola Kvist, Johan Sanmartin Berglund, Sandra Diaz Ruiz, Martin Boldt, Carl-Erik Flodmark, Peter Anderberg

**Affiliations:** 1 Department of Health Blekinge Institute of Technology Karlskrona Sweden; 2 Department of Pediatric Radiology Karolinska University Hospital Stockholm Sweden; 3 Department of Computer Science Blekinge Institute of Technology Karlskrona Sweden; 4 Department of Clinical Sciences Lund University Lund Sweden

**Keywords:** chronological age assessment, bone age, skeletal maturity, machine learning, magnetic resonance imaging, radius, distal tibia, proximal tibia, distal femur, calcaneus

## Abstract

**Background:**

Bone age assessment (BAA) is used in numerous pediatric clinical settings as well as in legal settings when entities need an estimate of chronological age (CA) when valid documents are lacking. The latter case presents itself as critical as the law is harsher for adults and granted rights along with imputability changes drastically if the individual is a minor. Traditional BAA methods have drawbacks such as exposure of minors to radiation, they do not consider factors that might affect the bone age, and they mostly focus on a single region. Given the critical scenarios in which BAA can affect the lives of young individuals, it is important to focus on the drawbacks of the traditional methods and investigate the potential of estimating CA through BAA.

**Objective:**

This study aims to investigate CA estimation through BAA in young individuals aged 14-21 years with machine learning methods, addressing the drawbacks of research using magnetic resonance imaging (MRI), assessment of multiple regions of interest, and other factors that may affect the bone age.

**Methods:**

MRI examinations of the radius, distal tibia, proximal tibia, distal femur, and calcaneus were performed on 465 men and 473 women (aged 14-21 years). Measures of weight and height were taken from the subjects, and a questionnaire was given for additional information (self-assessed Tanner Scale, physical activity level, parents' origin, and type of residence during upbringing). Two pediatric radiologists independently assessed the MRI images to evaluate their stage of bone development (blinded to age, gender, and each other). All the gathered information was used in training machine learning models for CA estimation and minor versus adult classification (threshold of 18 years). Different machine learning methods were investigated.

**Results:**

The minor versus adult classification produced accuracies of 0.90 and 0.84 for male and female subjects, respectively, with high recalls for the classification of minors. The CA estimation for the 8 age groups (aged 14-21 years) achieved mean absolute errors of 0.95 years and 1.24 years for male and female subjects, respectively. However, for the latter, a lower error occurred only for the ages of 14 and 15 years.

**Conclusions:**

This study investigates CA estimation through BAA using machine learning methods in 2 ways: minor versus adult classification and CA estimation in 8 age groups (aged 14-21 years), while addressing the drawbacks in the research on BAA. The first achieved good results; however, for the second case, the BAA was not precise enough for the classification.

## Introduction

### Background

Skeletal maturity is a radiological concept that refers to the stage of bone development in an individual [1]. This maturation process occurs gradually in the growth plates and is measured by the degree of mineralization of the bone along with its size and shape [1]. Bone age (BA) is a closely related concept in which age is estimated based on the degree of skeletal maturity of an individual [2].

The estimation of the BA of an individual, or bone age assessment (BAA), is performed in numerous clinical settings involving diagnosis and time of treatment of orthopedics, orthodontics, endocrinology, growth disorders, and estimations of final height [3]. In these cases, the BA of an individual is assessed by medical professionals and compared with their chronological age (CA). If they are found to be relatively advanced or retarded, appropriate actions are taken by the medical professionals.

BAA is also performed outside the clinical setting when legal entities need an estimation of the CA of an individual for judicial decisions when valid documents are lacking. This refers to cases regarding adoption, criminal proceedings, and pedopornography judicial issues as well as in determining age fraud in youth sports competitions [4-7]. Furthermore, with the upsurge of immigration due to the rise of worldwide conflicts, another critical scenario in which BAA is applied concerns the determination of an individual being minor in the absence of valid or trustworthy documents. This is the case of numerous young asylum seekers who are given special rights granted by the United Nations Convention on the Rights of the Child, regarding reception, health care, and education [8,9].

From these examples, it is possible to assume that, especially regarding legal standpoints, BAA is a crucial tool for making high stake decisions that have the potential to greatly affect individuals’ lives.

### Traditional BAA

The traditional methods for BAA are based on the appearance of growth plates through the analysis of diaphysis (primary ossification centers) and epiphysis (secondary ossification centers), where cartilage tissue gradually turns into bone tissue during the process of bone development. A process that ceases when the diaphysis and epiphysis are fused, indicating that the growth plate is ossified [1].

The most common procedures for BAA are the Greulich-Pyle (GP) and Tanner-Whitehouse (TW) methods. Both of these methods assess radiographic images of the hand and wrist areas as these are regions of interest (ROIs) with a large number of ossification centers aggregated in a small area that can easily have images taken from.

The GP method [10] attributes BA by comparing the radiograph image of the individual being assessed to the nearest reference image in a hand and wrist atlas in terms of bone development. The TW method [11] is a scoring system that evaluates the ulna, radius, carpals, and 13 short bones of the hand. Scores are attributed to these regions based on the stage of bone development, which ranges from A to I. The scores are then aggregated in a total score that is converted into the BA.

Having been developed in the 30s and 50s, the GP and TW methods, respectively, conveyed groundbreaking developments in numerous clinical settings and are still heavily employed for BAA purposes to this day.

### Other Proposed BAA Methods

The field of BAA evolved as the GP and TW methods were proposed, exploring new ROIs with different ossification timings. This section summarizes the proposed studies regarding BAA in various ROIs.

Newer hand and wrist studies on BAA include the Gilsanz and Ratib [1] digital hand atlas and the Fels method [12]. The first is composed of artificially created reference images that represent the average development of 29 classes of subjects aged from 0 to 18 years. The Fels method [12] is a statistical method that provides a relative measure of the BA and standard error that takes into consideration the distribution of chronological ages in the study’s sample with BA similar to the individual being assessed. It is based on 98 indicators of bone maturity (ossification, radiopaque densities, bony projection, shape changes, and ossification of epiphysis).

Clavicle staging systems observe one or both sides of the medial clavicular epiphysis. The method proposed by Kreitner et al [13] presents 4 stages of ossification of the medial clavicular epiphysis, in which the last stage may have an epiphyseal scar visible. Schmeling et al [9] proposed 5 stages of ossification, but the last stage was only achieved when the epiphyseal scar was not apparent. Kellinghaus et al [14] built on the Schmeling et al [9] staging by applying subclassifications for the second and third stages. These studies report complete ossification of this growth plate around the ages of 26 to 27 years.

Knee studies proposed staging systems that also vary on subscales on specific stages and the appearance of the epiphyseal scar in the last stage. O’Connor et al [15] proposed 5 stages of ossification of the distal femur, proximal tibia, and proximal fibula epiphysis (the epiphyseal scar may be visible in the last stage). Dedouit et al [16] proposed 5 stages of ossification of the distal femur and proximal tibia epiphysis, assessing the appearance of cartilage signal intensity with magnetic resonance imaging (MRI). Krammer et al [17] proposed 5 stages of ossification of the distal femur epiphysis, with subclassifications on the second and third stages, with the last stage achieved only when the epiphyseal scar is no longer visible. This method also makes use of MRI images. Knee studies usually argue that a subject is younger or older than the age of 18 years.

Studies on foot ROIs are usually concerned with younger ages. Ekizoglu et al [18] proposed a staging system for the foot ROI that shows complete ossification in the ages between 12 and 16 years.

Not very much is explored in the literature, the arm ROI was studied in the proximal humerus epiphysis by Ekizoglu et al [19] employing a scoring system based on Schmeling et al [9] and Kellinghaus et al [14] on MRI images. This study points out the earliest ages for the last stage of ossification at 17 and 18 years.

### Drawbacks in Assessing Chronological Age Using BAA Methods

In the lack of valid or trustworthy documents, BAA is currently employed as a valuable tool for legal entities to evaluate CA with regard to important legal ages. Nevertheless, it is possible to identify several drawbacks of the largely employed GP and TW methods as well as recently proposed methods, regarding the use of BAA for CA determination:

- They almost exclusively employ medical imaging techniques that expose the individual to ionizing radiation, such as radiographs, which raises grave ethical issues especially with regard to exposing minors to radiation for nontherapeutic purposes.
- They only focus on the physical appearance of the growth plates, not including other information that might possibly affect bone development [20].
- They mostly focus on a single ROI, which in the vast majority of cases is the hand area [20].

The first drawback can be addressed by the employment of MRI technology, which is already present in some of the mentioned knee and arm studies. Besides being a radiation-free modality of medical imaging, it also allows the manipulation of contrast to highlight different tissue types [21]. The epiphyseal plate consists of cartilage tissue, which is mainly composed of collagen fiber protein. Collagen has a 3D structure of fibers that, in MRI images, is shown as zones of different intensities, giving it a multilaminar appearance. It is known that the structure of cartilage changes in terms of the number of laminae and thickness in the course of bone development [22]. Hence, contrary to radiographs that highlight the bone, the MRI technology might have the potential to offer better visualization of growth plates, thus being an interesting radiation-free modality of medical imaging for BAA.

To address the second drawback, the methods for assessing BA should investigate factors that may play a role in the process of bone development and ossification of growth plates, that is, BMI [20,23], pubertal growth [24], physical activity [25], ethnicity [8,20,26], and socioeconomic factors [8], which are often overlooked [20].

Addressing the third drawback could be done by employing multiple ROIs. When it comes to estimations of CA, most of the BAA studies, especially methods that propose stages of maturity for set ROIs, follow an approach of identifying the minimum age in which the ossification of the growth plate is completed for a particular ROI. These studies usually focus on a single age of legal importance, which varies significantly between countries, with ages ranging from 14 to 21 years [13]. Using multiple ROIs may provide more information about more ages.

An additional drawback that is specific to the GP and TW methods is that they are based on data collected from subjects of average and upper socioeconomic classes in the 30s and 50s, respectively. Hence, these methods may not reflect secular trends that nowadays point to higher height and earlier puberty [27], which could affect the accuracy of the methods. For the TW method, an update released in 2001 (TW3) revised the calculation of the BA from the attributed scores to address this problem [28].

### Machine Learning for BAA

From the presented drawbacks, it is noticeable that the BAA research could benefit from methods that are able to aggregate multiple pieces of information (ie, multiple ROIs and factors) in a systematic way. A technology that is able to work in this setting is machine learning (ML), which is already widely employed in diverse medical fields, such as diabetes, cancer, cardiology, mental health, and the analysis of clinical text data [29,30]. ML consists of various types of algorithms that are able to learn how to perform a task from a set of examples while improving its performance based on its experience in carrying out a particular task. It builds a model that encapsulates the knowledge to perform the task; then, in light of new data, the model is able to correctly perform the learned task within an acceptable measure of performance [31].

ML algorithms have already been employed in various models for assessing the BA of an individual. A recent systematic literature review on BAA with ML methods [20] showed that the research is heavily focused on models that make use of a single ROI, the hand in most cases, having radiographs as the choice of imaging technology and do not usually consider other factors that could play a role in bone development [20]. The most notable, commercially available ML BAA system is the BoneXpert [32], which performs an automatic radiograph analysis based on the GP and TW methods. However, it covers the age range of 2 to 17 years and leaves out important legal ages.

### Objectives of the Study

Given the importance of the assessment of CA through BAA in numerous scenarios and its potential ways of affecting the lives of young individuals, it is important to focus on the drawbacks of the methods currently in use and investigate the potential of BAA in estimating CA. Thus, the objectives of this study are as follows:

- To investigate the extent to which ML models can aid in CA estimation through BAA in young individuals aged 14 to 21 years.
- To investigate whether ML models can aid in the determination of minors through BAA, considering the threshold of 18 years, in young individuals aged 14 to 21 years.
- To address the drawbacks in the research on CA estimation from BAA, with regard to using radiation-free medical imaging technology, the assessment of multiple ROIs and other factors that may play a role in bone development.

## Methods

### Overview

To train the CA estimation ML models proposed in this paper, MRI images of the wrist, knee, and foot were taken from volunteer subjects and assessed by radiologists to evaluate their stage of bone development. The 5 growth zones considered in this study were calcaneus, distal tibia, proximal tibia, distal femur, and radius. Each growth zone was assessed separately and blinded to gender and age.

Before the examination, the subjects had their height and weight measured for the BMI calculation and were asked to answer a questionnaire to gather information on their physical activity level, parents’ origin, type of residence during upbringing, and a self-assessed Tanner Scale of pubertal growth [33,34].

All radiological and nonradiological data gathered were used to train binary and multiclass classifiers. For the binary classifier, the individuals in the sample were divided into minors or adults, with a threshold of 18 years, and the classification followed into discriminating individuals into 1 of the 2 classes. The multiclass classifier aims to classify an individual into 1 of the 8 classes defined by age groups ranging from 14 to 21 years.

The remainder of this section details the population, data used in the experiments, statistical analysis, and procedures for model building in the experiments.

### Recruitment

This study prospectively conducted MRI examinations of 938 healthy subjects (465 males and 473 females) aged between 14 and 21 years (inclusive), during 2017 and 2018. The participants of the study had images taken from the knee, foot, and wrist in the same examination session. Additionally, the weight and height of each participant were also collected to calculate the BMI.

The following criteria were used to determine participation in the study:

- Inclusion criteria: the participants should have been born in Sweden, where the study was conducted, and have a birth certificate verified by the Swedish national authorities.
- Exclusion criteria: a history of bilateral fractures or trauma near the regions of assessment, a history of chronic disease or the use of long-term medications, noncompliance during the examination, having resided outside Sweden for more than six consecutive months, or past or current pregnancy (all female subjects were tested).

### Data Privacy and Study Ethics

The study was conducted in accordance with the Declaration of Helsinki and was approved by the Central Ethical Review Board in Stockholm (diary numbers: 2017/4-31/4, 2017/1184-32, 2017/1773-32). Written informed consent was obtained from all subjects and legal guardians (in the case of subjects aged younger than 18 years). All data were anonymized and stratified by age and gender.

### Population

A total of 455 male and 467 female subjects constituted the final sample (Table 1). After the MRI examinations and assessment of images by radiologists, 10 male and 6 female subjects were removed from the study’s sample because they had the assessment of one or more ROI missing. The missing values for the assessment by the radiologists could be due to one of the following reasons: movement artifact, error in the sequence that made the image nongradable, likely trauma in the region of assessment, and missing MRI examination in one or more ROIs.

**Table 1:** Demographics of the final sample.

Demographics	Age group								Total	
	14	15	16	17	18	19	20	21		
Number of female subjects	59	58	57	60	59	57	57	60		467
Number of male subjects	58	56	60	58	53	58	53	59		455

### Data and Data Collection Procedures

The data used to train the classifiers were the radiologists’ assessment of the calcaneus, distal tibia, proximal tibia, distal femur, and radius growth zones; the additional information gathered before the examination was physical activity level, parents’ origin, type of residence during upbringing, and a self-assessed Tanner Scale of pubertal growth and BMI. The following section details the data and procedures for collection.

#### MRI Examinations

MRI examinations were performed to capture images of the calcaneus, distal tibia, proximal tibia, distal femur, and radius growth plates of the subjects participating in the study. All MRI examinations were conducted within 6 months of the subjects’ birthday date on 1.5-T whole-body MRI scanners with dedicated hand, knee, and ankle coils. The examinations were performed on the nondominant side of the knee, hand, and foot, save when past fracture or trauma had taken place near the region. In these cases, the dominant side was imaged. The images of all ROIs were taken in the same examination session.

The examinations were carried out at 2 sites. Site 1 used Magnetom Avanto Fit (Siemens Healthcare GmbH) and Achieva (Philips Healthcare) whole-body scanners, and Site 2 used a Signa (GE Healthcare) whole-body scanner. All examinations followed the same protocol, which included a T2 sequence with cartilage dedicated exposure. The settings were 256×256 pixel resolution and 160×160 mm field of view.

#### Assessment of Magnetic Resonance Images

The assessment of the MRI images was performed independently by 2 radiologists with 3 and 30 years of experience in pediatric radiology, who were blinded to the age and gender of the participants. A third radiologist with 13 years of experience in pediatric radiology assessed the images when the first 2 radiologists could not reach a final agreement about the stage.

The staging system used to assess MRI images is a version of the staging methods proposed by Dedouit et al [16] and Kellinghaus et al [14] with minor modifications. This staging is defined as follows:

- Stage 1: Continuous, stripe-like, cartilage signal intensity is present between the metaphysis and epiphysis with a thickness greater than 1.5 mm with a multilaminar appearance.
- Stage 2: Continuous cartilage signal intensity is present between the metaphysis and epiphysis with a thickness greater than 1.5 mm with increased signal intensity but without a multilaminar appearance.
- Stage 3: Continuous cartilage signal intensity is present between the metaphysis and epiphysis with a thickness of less than 1.5 mm with increased signal intensity.
- Stage 4a: Noncontinuous cartilage signal intensity. A hazy area involving one-third or less of the growth plate is present between the metaphysis and epiphysis, representing the epiphyseal-metaphyseal fusion.
- Stage 4b: Noncontinuous cartilage signal intensity. A hazy area involving between one-third and two-third of the growth plate is present between the metaphysis and epiphysis, representing epiphyseal-metaphyseal fusion.
- Stage 4c: Noncontinuous cartilage signal intensity A hazy area involving more than two-thirds of the growth plate is present between the metaphysis and epiphysis, representing epiphyseal-metaphyseal fusion.
- Stage 5: The epiphyseal cartilage fused completely with or without an epiphyseal scar in all MRI slices.

#### Body Mass Index

The BMI was calculated using the measures of the participants’ weight *w* and height *h*, as in the following equation 1 [35]:

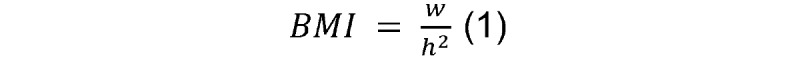



Data characteristics regarding the calculated BMI for the subjects are shown in the Multimedia Appendix 1.

#### Questionnaire Information

Additional information from the participants was gathered by a questionnaire given to them at the examination session. The information gathered by the questionnaire refers to the variables “Residence,” “Physical Activity,” “Parent Origin,” and “Tanner Scale,” shown in Table 2, which summarizes all input and output variables considered for building the models. Data characteristics regarding the data collected by the questionnaire are shown in the Multimedia Appendix 1.

**Table 2:** Summary of the input and output variables considered in the model building.

Variable		Description	Values
**Input variables**			
	Radius	Radiologists’ assessments of the Radius growth zone	Stage 1; Stage 2; Stage 3; Stage 4a; Stage 4b; Stage 4c; Stage 5
	Distal femur	Radiologists’ assessments of the distal femur growth zone	Stage 1; Stage 2; Stage 3; Stage 4a; Stage 4b; Stage 4c; Stage 5
	Proximal tibia	Radiologists’ assessments of the proximal tibia growth zone	Stage 1; Stage 2; Stage 3; Stage 4a; Stage 4b; Stage 4c; Stage 5
	Distal tibia	Radiologists’ assessments of the distal tibia growth zone	Stage 1; Stage 2; Stage 3; Stage 4a; Stage 4b; Stage 4c; Stage 5
	Calcaneus	Radiologists’ assessments of the calcaneus growth zone	Stage 1; Stage 2; Stage 3; Stage 4a; Stage 4b; Stage 4c; Stage 5
	BMI	Body mass index of the participant, calculated as in the equation (1)	Numeric
	Residence	Type of residence the participant lives in (or lived during upbringing)	Rented; owned
	Physical activity	The participants’ daily level of activity	Highly inactive; inactive; little active; active; highly active
	Parent origin	Origin of the participants’ parents, regarding if they were born outside Sweden or not	No foreign-born parents; one foreign-born parent; both foreign-born parents
	Tanner scale	Self-assessed Tanner Scale for pubertal growth [33,34]	Stage 1; Stage 2; Stage 3; Stage 4; Stage 5
**Output variables**			
	Minor	Characterizes the participant as being a minor or not, regarding the threshold of 18 years. This is the output variable for the binary classification models	Yes; no
	Age	Regards the age group which the participant belongs to. This is the output variable for the multi-class classification models	14; 15; 16; 17; 18; 19; 20; 21

### Statistical Analyses

The Cohen kappa coefficient [36] and percent of agreement [37] were calculated to measure the interobserver agreement between the pediatric radiologists in all investigated ROIs. Statistical analyses were performed using SPSS Statistics (version 24; IBM Corp).

### Model Building

#### CA Estimation Models

In this study, various ML algorithms were investigated to build classifiers to discriminate subjects into minor (positive class) or adults (negative class) and classifiers to classify subjects into 1 of 8 age groups (14 to 21 years). Models for male and female subjects were built separately.

#### Data Preprocessing

The data used to build the models consisted of the radiologists’ assessment of the 5 growth zones, following the aforementioned stages, the questionnaire information, and the calculated BMI. These data presented missing values that were handled by the K-nearest neighbor (KNN) multiple imputations. This technique finds K complete entries that are the closest to an incomplete entry (ie, contains missing data) and fills its missing values with the mean (in the case of numeric variables) or the most frequent one (in the case of categorical variables) [38]. In this study, the number of nearest neighbors K for the KNN imputation was set to 1. The motivation for this choice is based on literature findings that advise limiting K as a way to preserve the original variability of the data, reducing the risk of entries having few neighbors that are too distant from each other [39]. There is also a risk of increasing the influence of noise in the data with a small K, but as in the data set of this study, the highest rate of imputed instances was 1.9%; this influence was considered to be not very relevant. The distance used by the KNN multiple imputation technique was the Gower distance [40]. The number of imputed instances for each variable in both male and female subsets is shown in Table 3.

**Table 3:** Number of imputed instances and percentage over the male and female data sets.

Variable	Male data set, n (%)	Female data set, n (%)
Radiologists' assessments of the radius, distal femur, proximal tibia, distal tibia, calcaneus	0 (0)	0 (0)
Residence	1 (0.2)	3 (0.6)
Physical activity	9 (1.9)	6 (1.2)
Tanner Scale	3 (0.6)	1 (0.2)
BMI	0 (0)	0 (0)
Parents origin	0 (0)	3 (0.6)

#### ML Algorithms

The choice of the ML algorithms explored in this study was based on the summary of the evidence of a recently published systematic literature review (SLR) on the application of ML for BAA [20]. This SLR points out that the studies proposing BAA classifiers employ algorithms of the following categories: artificial neural networks, support vector machines, Bayesian networks, decision trees, and K-nearest neighbors. An additional search was conducted in the literature (Scopus, PubMed, and Web of Science), after the search date of the mentioned SLR [20] (February 2019) to look for additional algorithms, but no new categories were found to be added to the list.

Another motivation for this choice of ML algorithms is that it also guarantees a diversified list of classifiers that make use of different types of learning techniques, such as rule-based, instance-based, Bayesian inference, kernel, and perceptron learners. We referred to the following book by Kuhn and Johnson [41] for the specific algorithms and implementations used in this study.

Therefore, the choice of ML algorithms for the experiments of this study includes decision tree, random forest, multilayer perceptron, support vector machines, naïve Bayes, and K-nearest neighbors.

#### Experimental Setup

All experiments were performed using a stratified, nested cross-validation [42]. In this approach, in each iteration, one fold of the outer cross-validation is used for testing and the remaining 4 are used in an inner cross-validation to tune the algorithm’s hyperparameters. This was done to obtain a more reliable estimate of the error as the test fold in each outer iteration is not used to execute performance optimization [43]. It is also worth noting that the data splits were performed in a stratified manner, which means that the classes’ proportions in each split are kept the same as in the total sample. In the experiments of this study, a five-fold outer, three-fold inner stratified nested cross-validation was performed. The reduced number of folds in the inner cross-validation was employed to avoid having a low number of subjects to represent each class in the folders, due to the high number of classes in the multiclass classification problem. Additionally, before each inner cross-validation, a grid search was performed to find suitable hyperparameters for each of the selected ML algorithms. The hyperparameters for each selected algorithm are listed in Table 4. The ML experiments were conducted in the R framework with the *caret* package. The default versions of the algorithms were used.

**Table 4:** Configuration of the R algorithms included in the experiment.

ML^a^ algorithm	R implementation	Tuning parameters
Decision tree	*rpart*	*cp*
Random forest	*rf*	*mtry*
Multi-layer perceptron	*mlp*	*size*
Support vector machines	*svmRadial*	*Sigma, C*
Naïve Bayes	*nb*	*fL, usekernel, adjust*
K-nearest neighbors	*knn*	*k*

^a^ML: machine learning.

### Model Evaluation Metrics

The performance metrics used to evaluate the models were as follows: mean absolute error (MAE), root mean squared error (RMSE), accuracy, precision, recall, and area under the curve (AUC), as in Gaudette and Japkowicz [44] and Sokolova and Lapalme [45] guidelines for ordinal multiclass classification. For the binary classification models, all but MAE and RMSE are used. The SDs for each metric are also reported.

The MAE represents the mean of the absolute difference between the estimated age output of the classifier and the correct CA of the subject, over all examples. The RMSE gives more weight to larger errors compared with MAE, which tends to prefer fewer errors overall. The MAE and RMSE are calculated as follows in equations 2 and 3, respectively:

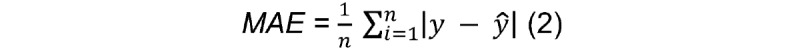

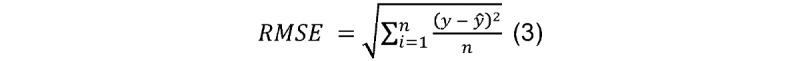





Where n is the number of samples, 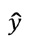 is the estimated age, and y is the CA of the subject.

For the remaining evaluation metrics, considering l the number of classes, we define the following:

- True-positives (TP): Entries predicted to be in class C_l_ actually in class C_l_.
- False-positives (FP): Entries predicted to be in C_l_ but are not actually in class C_l_.
- True-negatives (TN): Entries not predicted to be in C_l_ and are not actually in class C_l_.
- False-negatives (FN): Entries not predicted to be in C_l_, but are actually in class C_l_.

The accuracy, precision, recall, and AUC for binary classification are calculated as follows:

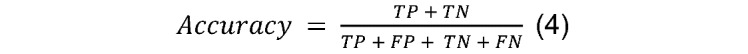

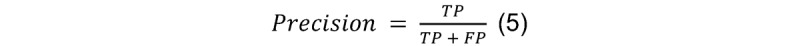

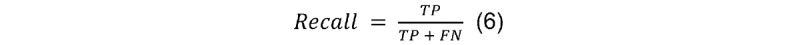

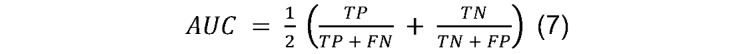









In the case of the multiclass classification, these are calculated as the average of the metrics calculated for each class C_l_ (macro averaging) [45]. The AUC metric is calculated by averaging pairwise comparisons, as proposed by Hand and Till [46].

General results are given for the ML algorithms in terms of the mean and SDs of each of the performance metrics for the outer cross-validation test sets. In-depth results are given to the best performing models.

## Results

### Interobserver Agreement

The kappa Cohen coefficient was calculated to evaluate the agreement between the 2 observers’ assessments of the MRI images. The results indicated substantial agreement according to the general guidelines [47] for all of the assessed ROIs: 0.77 for the calcaneus, 0.65 for the distal femur, 0.72 for the distal tibia, 0.73 for the proximal tibia, and 0.67 for the radius.

The percent agreement for the assessed ROI was as follows: 94.2% for the calcaneus, 80.8% for the distal femur, 90.6% for the distal tibia, 86.8% for the proximal tibia, and 79.4% for the radius. These results show that the radiologists agreed on a stage in the vast majority of cases.

### Results of the Growth Plate Assessments

The results of the assessments of the calcaneus, distal tibia, proximal tibia, distal femur, and radius for male and female subjects are shown in detail in Multimedia Appendices 2 and 3, respectively.

In all of the assessed growth plates, for both sexes, stages 1 and 2 were not evidenced. Few instances of stage 3 were observed on male subjects on the calcaneus and radius growth plates, accounting for 2 and 15 cases, respectively. In female subjects, stage 3 was evidenced in only 2 cases for the radius growth plate.

The female subjects’ results show that for all assessed growth plates, nearly all or most of the sample was already in the last stage of ossification (stage 5): 94.6% of the calcaneus, 90.8% of the distal tibia, 81.6% of the proximal tibia, 74.5% of the distal femur, and 65.5% of the radius cases. These numbers moderately change for male subjects, accounting for 80.4% of the calcaneus, 70.1% of the distal tibia, 57.6% of the proximal tibia, 54.9% of the distal femur, and 47.4% of the radius cases.

Table 5 shows the proportion within each age group of subjects who had all of the growth plates considered in this study already in stage 5. This table shows that female subjects had all growth plates fused 2 years before the male subjects. For female subjects, from the age of 19 years, all subjects of the sample already have all of the growth plates fused, although for male subjects, the same happens from the age of 21 years.

**Table 5:** Numbers and percentages (over each age group) of subjects with all of the growth plates in stage 5, for male and female subjects.

Characteristic		Female subjects, n (%)	Male subjects, n (%)
**Age group (years)**			
	14	2 (3.3)	0 (0)
	15	8 (13.7)	0 (0)
	16	23 (40.3)	3 (5)
	17	44 (73.3)	13 (22.4)
	18	53 (89.8)	31 (58.4)
	19	57 (100)	50 (86.2)
	20	57 (100)	50 (94.3)
	21	60 (100)	59 (100)
Total		304 (65.1)	206 (45.2)

### Results for the Classification of Minors Versus Adults

A threshold of 18 years was used to determine adulthood in the classification of minors versus adults, which is the case in many European countries. MAE and RMSE were not used as performance metrics in this case because for classifications they only make sense in the context of an ordinal classification. The results for the male subjects’ binary classifiers in terms of the mean and SD of the performance metrics on the outer cross-validation test sets are shown in Table 6.

**Table 6:** Mean performance metrics and respective SDs (in years) for the classification of minor versus adults for the male subjects.

Types	Accuracy, mean (SD)	AUC^a^, mean (SD)	Precision, mean (SD)	Recall, mean (SD)
Decision tree	0.90 (0.02)	0.90 (0.02)	0.86 (0.04)	0.96 (0.03)
Random forest	0.90 (0.01)	0.90 (0.01)	0.87 (0.03)	0.94 (0.04)
Support vector machines	0.90 (0.02)	0.90 (0.02)	0.87 (0.04)	0.93 (0.07)
Multi-layer perceptron	0.82 (0.17)	0.82 (0.16)	0.79 (0.16)	0.95 (0.04)
K-nearest neighbors	0.87 (0.02)	0.87 (0.02)	0.84 (0.03)	0.92 (0.03)
Naïve bayes	0.73 (0.04)	0.74 (0.04)	0.65 (0.03)	1.00 (0.00)

^a^AUC: area under the curve.

The decision tree, random forest, and support vector machine algorithms had very similar results in general, presenting no significant difference between them. The random forest algorithm was chosen in terms of the best combination of precision and recall, but in practical settings, there are no differences between these algorithms. Table 7 shows the random forest results for each of the outer cross-validation test sets. The average model was chosen in terms of median accuracy, which was 0.90. Between Models 1 and 4, Model 1 was chosen for better recall in classifying minors. The optimized hyperparameter given by the grid search for Model 1 was *mtry=2* (number of candidate variables at each tree split)*.*

**Table 7:** Performance results for the Random Forest algorithm on each of the outer cross-validation test sets, for the male sample.

Model	Accuracy	AUC^a^	Precision	Recall—minors	Recall—adults
1 (median)	0.90	0.90	0.83	1.00	0.80
2	0.89	0.89	0.87	0.91	0.87
3	0.89	0.89	0.87	0.96	0.83
4	0.90	0.90	0.90	0.89	0.91
5	0.92	0.92	0.89	0.96	0.89

^a^AUC: area under the curve.

**Figure 1:**
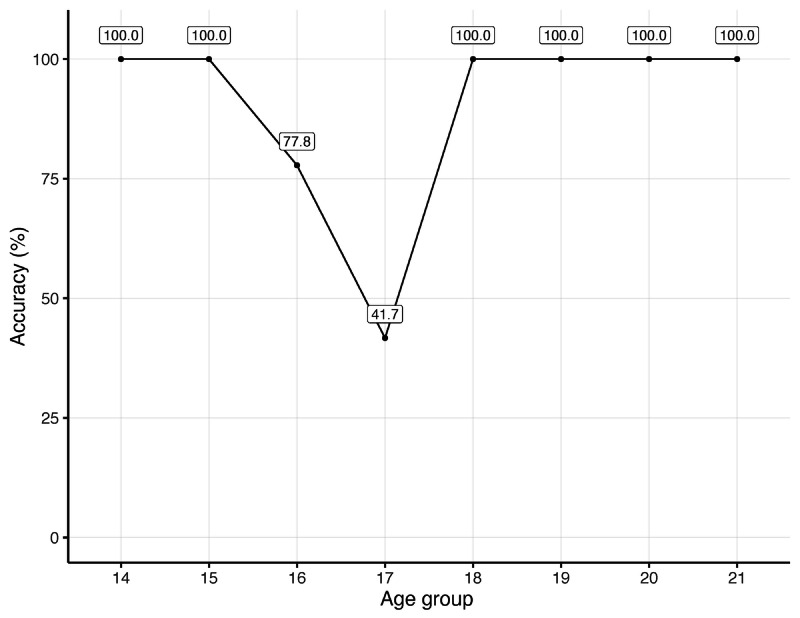
Accuracy per age group for the minor versus adults classification model, for male subjects.

Figure 1 presents the results achieved by Model 1 per age group. It is important to note that even with low accuracy results for the age of 17 years (41.7%), the model still minimizes the error of classifying minors as adults, achieving a recall of 100% for this classification.

In the case of the female subjects, the decision tree, random forest, and multi-layer perceptron algorithms presented very similar results (Table 8), which do not present a relevant significant difference between them. The chosen algorithm for the female subject case was the random forest algorithm for the best combination of performance measures. Table 9 shows the results for the random forest algorithm in each of the outer cross-validation test sets. Except for Model 1, there was essentially no relevant variation between models, and in practical settings, they can be considered equal. Thus, Model 2 was chosen as the average model. The optimized hyperparameter given by the grid search for Model 1 was mtry=6.

**Table 8:** Mean performance metrics and respective SDs (in years) for the classification of minor versus adults for the female subjects.

Types	Accuracy, mean (SD)	AUC^a^, mean (SD)	Precision, mean (SD)	Recall, mean (SD)
Decision tree	0.82 (0.02)	0.82 (0.02)	0.74 (0.02)	0.97 (0.01)
Random forest	0.83 (0.02)	0.83 (0.01)	0.76 (0.02)	0.97 (0.01)
Support vector machines	0.81 (0.04)	0.81 (0.04)	0.75 (0.04)	0.92 (0.05)
Multi-layer perceptron	0.82 (0.02)	0.82 (0.02)	0.75 (0.02)	0.95 (0.04)
K-nearest neighbors	0.78 (0.06)	0.78 (0.06)	0.73 (0.06)	0.87 (0.08)
Naïve bayes	0.67 (0.03)	0.67 (0.02)	0.60 (0.02)	1.00 (0.00)

^a^AUC: area under the curve.

**Table 9:** Performance results for the random forest algorithm on each of the outer cross-validation test sets, for the female sample.

Model	Accuracy	AUC^a^	Precision	Recall—minors	Recall—adults
1	0.81	0.81	0.73	0.96	0.66
2 (median)	0.84	0.84	0.77	0.96	0.72
3	0.84	0.84	0.77	0.98	0.70
4	0.84	0.84	0.78	0.96	0.72
5	0.84	0.84	0.77	0.98	0.70

^a^AUC: area under the curve.

**Figure 2:**
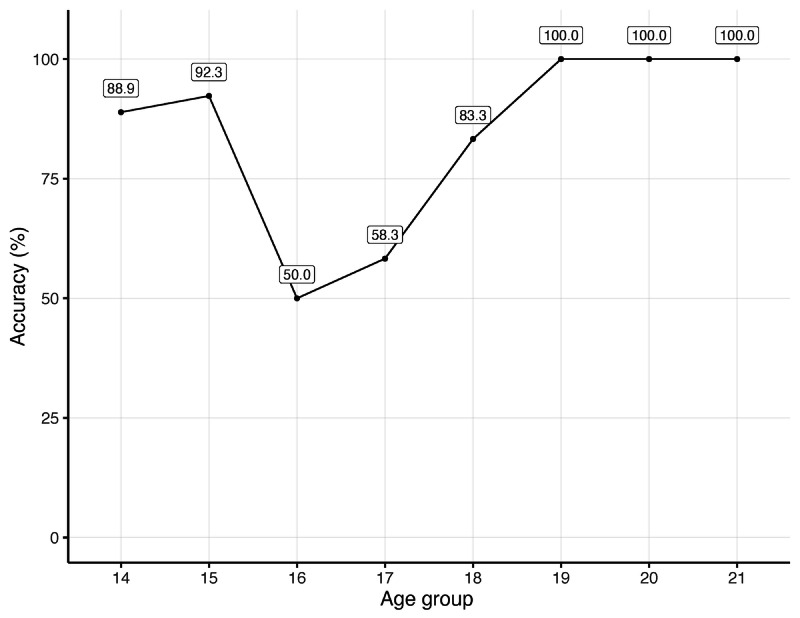
Accuracy per age group for the minor versus adults classification model, for female subjects.

The accuracies per age group are shown in the graph in Figure 2. The model achieved lower accuracies for the ages of 16 and 17 years (50.0% and 58.3%, respectively), but as in the case of the male subjects, the model minimizes the worst type of error, which is the misclassification of minors, achieving a high recall of 96%.

### Results for the CA Estimation Models

The CA estimation models are multiclass classifiers that aim to classify subjects in 1 of the 8 age groups (from 14 to 21 years). Table 10 shows the results for the male subjects’ models in terms of the mean and SDs of the performances on the outer cross-validation test sets. The best performing algorithm in the male case was the multilayer perceptron (MLP), which achieved the best MAE (0.98 years), mean RMSE (1.32 years), and mean precision (0.65 years) values, in addition to having the second-best values of mean accuracy and mean AUC.

**Table 10:** Mean (SD) of the performance metrics for the male subjects’ classification models.

Algorithms	MAE^a^ (years), mean (SD)	Accuracy, mean (SD)	RMSE^b^ (years), mean (SD)	AUC^c^, mean (SD)	Precision, mean (SD)	Recall, mean (SD)
Decision tree	1.28 (0.13)	0.32 (0.03)	1.78 (0.17)	0.81 (0.02)	0.49 (0.06)	0.81 (0.11)
Random forest	1.04 (0.07)	0.34 (0.03)	1.44 (0.13)	0.85 (0.01)	0.57 (0.09)	0.73 (0.14)
Support vector machine	1.03 (0.09)	0.34 (0.03)	1.43 (0.08)	0.85 (0.01)	0.52 (0.09)	0.67 (0.12)
Multi-layer perceptron	0.98 (0.08)	0.33 (0.02)	1.32 (0.13)	0.84 (0.01)	0.65 (0.27)	0.61 (0.31)
K-nearest neighbor	1.16 (0.11)	0.30 (0.04)	1.57 (0.15)	0.82 (0.03)	0.59 (0.10)	0.59 (0.10)
Naïve bayes	1.07 (0.10)	0.29 (0.02)	1.39 (0.19)	0.81 (0.01)	0.57 (0.06)	0.58 (0.21)

^a^MAE: mean absolute error.

^b^RMSE: root mean squared error.

^c^AUC: area under the curve.

The performances of the MLP algorithm on each of the outer cross-validation test sets are shown in Table 11. The average model was chosen in terms of the median MAE, which corresponds to Model 1, with a value of 0.95 years. The optimized hyperparameter given by the grid search for the average MLP model was *size=27* (number of units in the hidden layer). The average model was chosen to select an algorithm that would not be overly optimistic in its estimation.

**Table 11:** Performance results for the multi-layer perceptron algorithm on each of the outer cross-validation test sets, for the male sample.

Model	MAE^a^ (years)	Accuracy	AUC^b^	RMSE^c^ (years)	Recall	Precision
1 (median)	0.95	0.33	0.83	1.29	0.91	0.48
2	1.08	0.30	0.85	1.40	073	0.35
3	0.89	0.32	0.84	1.17	0.17	1.00
4	0.91	0.33	0.83	1.23	0.83	0.59
5	1.05	0.35	0.84	1.49	0.42	0.83

^a^MAE: mean absolute error.

^b^AUC: area under the curve.

^c^RMSE: root mean squared error.

The results for the chosen model, discriminated by age groups, are shown in Table 12. The model shows lower errors for the younger and older ages of the age spam considered in the study. In addition, the model has a clear trend of overestimating the ages of the male subjects in general. Thus, even with an MAE of 0.95 years, the model is limited to its capacity to classify individuals from the age of 16 years. From the age of 19 years, the model tends to classify all subjects as 20 years old as nearly all subjects of these ages have all growth plates on stage 5.

**Table 12:** Mean absolute error and SD for the average male model.

Measure	Age group							
	14	15	16	17	18	19	20	21
MAE^a^ (years)	0.18 (SD 0.60)	0.82 (SD 0.90)	1.25 (SD 1.44)	1.91 (SD 1.56)	1.50 (SD 1.45)	1.00 (SD 0.60)	0.00 (SD 0.00)	0.92 (SD 0.29)

^a^MAE: mean absolute error.

Table 13 shows the results for the CA estimation models for female subjects in terms of the mean and standard deviations of the performances on the outer cross-validation test sets. In the case of the female subjects, the best performing algorithm was the support vector machine (SVM), which achieved the best MAE (1.21 years), mean accuracy (0.32), mean RMSE (1.68 years), and mean AUC (0.80).

**Table 13:** Mean (SD) of the performance metrics, for the female subjects’ classification models.

Algorithms	MAE^a^ (years), mean (SD)	Accuracy, mean (SD)	RMSE^b^ (years), mean (SD)	AUC^c^, mean (SD)	Precision, mean (SD)	Recall, mean (SD)
Decision tree	1.31 (0.09)	0.28 (0.02)	1.78 (0.13)	0.80 (0.02)	0.56 (0.05)	0.82 (0.09)
Random forest	1.29 (0.10)	0.30 (0.03)	1.77 (0.10)	0.79 (0.02)	0.59 (0.13)	0.74 (0.17)
Support vector machine	1.21 (0.06)	0.32 (0.04)	1.68 (0.06)	0.80 (0.01)	0.55 (0.07)	0.71 (0.11)
Multi-layer perceptron	1.36 (0.24)	0.30 (0.02)	1.85 (0.37)	0.77 (0.02)	0.60 (0.11)	0.63 (0.22)
K-nearest neighbors	1.41 (0.12)	0.30 (0.02)	1.96 (0.12)	0.76 (0.03)	0.55 (0.07)	0.61 (0.18)
Naïve bayes	1.74 (0.23)	0.22 (0.02)	2.23 (0.27)	0.65 (0.03)	0.58 (0.06)	0.82 (0.09)

^a^MAE: mean absolute error.

^b^RMSE: root mean squared error.

^c^AUC: area under the curve.

Table 14 shows the performance results for each of the outer cross-validation test sets for the SVM algorithm. For the case of the female subjects, the median resulted in an MAE of 1.24 years, which pertained to Models 1 and 2. Model 1 was chosen as the average model for presenting the best accuracy between the two. The optimized parameter given by the grid search for the average SVM model was *sigma=0.0421* (kernel parameter) *and C=4* (penalty parameter).

The MAE results per age group are shown in Table 15. As in the male subjects’ case, the female model also overestimates the ages of female subjects in general, but with higher MAE and standard deviations.

**Table 14:** Performance results for the support vector machine algorithm on each of the outer cross-validation test sets, for the female sample.

Model	MAE^a^ (years)	Accuracy	AUC^b^	RMSE^c^ (years)	Recall	Precision
1 (median)	1.24	0.37	0.79	1.75	0.75	0.56
2	1.24	0.27	0.80	1.67	0.55	0.67
3	1.25	0.33	0.78	1.70	0.75	0.50
4	1.11	0.32	0.81	1.58	0.67	0.53
5	1.20	0.32	0.81	1.72	0.83	0.83

^a^MAE: mean absolute error.

^b^AUC: area under the curve.

^c^RMSE: root mean squared error.

**Table 15:** Mean absolute error and standard deviation for the male median model

Measure	Age group							
	14	15	16	17	18	19	20	21
MAE^a^ (years), mean (SD)	0.42 (0.79)	1.42 (1.93)	1.17 (1.79)	2.17 (2.49)	1.75 (1.88)	1.09 (1.43)	0.90 (1.27)	1.00 (1.34)

^a^MAE: mean absolute error.

## Discussion

### Principal Findings

This paper presents experiments with the estimation of CA and classification of minors versus adults (on the threshold of 18 years) of male and female subjects using ML algorithms. To build the models, 2 radiologists assessed the stage of bone development of the calcaneus, distal tibia, proximal tibia, distal femur, and radius growth plates of 455 male and 467 female volunteer subjects (922 subjects in total) from MRI images. Additional variables were also used to build the models: BMI, physical activity level, parents’ origin, type of residence during upbringing, and self-assessed Tanner Scale of pubertal growth. The methodology adopted in the study aimed at addressing the drawbacks of the BAA methods that are employed in CA estimation for legal scenarios.

From the stage assessments of the MRI images, we could infer that female subjects mature earlier than male subjects regarding the bone development of the knee, wrist, and foot, which is in line with prior studies [1,17,18,48,49]. In this study, the first age in which the whole sample had all fused growth plates (stage 5) was 19 years for female (467/467, 100%) and 21 years for male (455/455, 100%) subjects.

Another important point to be discussed with regard to the stage assessments is that the female sample had cases that had all of the considered growth plates already fused since the age of 14 years, spamming throughout all ages considered in the study (14 to 21 years). Since the assessment of stage 5, unlike the other stages, requires that all of the slices from the MRI examination to present a fused growth plate, even if there is a degree of misassessment, it would still mean that these cases would display a well-advanced level of maturation in all of these ages, implying a high degree of biological variation in the female sample with regard to BA. Additionally, in total, 65.5% (304/467) of the female sample consisted of cases in which the subjects presented all growth plates already in stage 5, which means that for classification purposes, for more than half of the sample, the estimation of CA would depend only on the additional factors (self-assessed Tanner Scale, BMI, residence type, physical activity, and parents origin), which were not enough to discriminate between age groups. This hindered the performance of classifiers, especially the CA estimation models. The same phenomenon occurred for the male sample, which also negatively affected the performance of the classifiers, but to a lesser degree, as 45.2% (206/455) of the sample had all growth plates of stage 5, from the age of 16 to 21 years.

The minors versus adults classification achieved good accuracy results for both male (90%) and female subjects (84%). These models portrayed a drop in the performance for the ages of 16 and 17; however, the recalls regarding the correct classification of minors were very high in both male and female cases (100% and 96%, respectively). This is important because the problem of minors versus adults classification is asymmetric as the misclassification of minors for adults in a judicial scenario is much more problematic than the inverse. In most cases, the application of the law is harsher for adults, and imputability, along with granted rights, can drastically change between these groups.

The CA estimation models achieved MAEs of 0.95 years and 1.24 years for male and female subjects, respectively. However, a look at a depth of the models showed that for both male and female models, only the ages of 14 and 15 years achieved acceptable MAE values. It could be argued that for the ages of 16 to 21 years, the estimation of a precise CA based on stages of bone development of the calcaneus, distal tibia, proximal tibia, distal femur, and radius growth plates would be somewhat unfit for male individuals and very unfit for female individuals. Furthermore, we could argue that staging may not offer a precise enough measure for the estimation of the CA of individuals of the ages considered in this study.

Compared with dental age, height, and age at menarche, BA is still the most reliable biological indicator for assessing maturation in young individuals [50], but it may not be a strong predictor of CA. BAA was conceived to be used in conjunction with CA to evaluate the maturation of an individual that can be delayed or advanced due to various factors that may include hormonal disorders, and chronic illnesses [8].

Regarding the agreement of the radiologists on the assessment of the growth plates' stage of development, substantial agreement was achieved, which is a satisfactory result as there is a lack of guidelines for BAA using MRI in the research. In addition, the individuals employed in the assessment of the MRI images were specialized pediatric radiologists with experience in BAA.

From a methodological point of view, this study employed a nested cross-validation approach that aims to avoid reporting overly optimistic results that could be derived from a *lucky* test set.

### Comparison With Prior Work

Most of the studies in the area of BAA that employ ML algorithms aim to build automatic approaches for estimating BA and evaluating BA given by radiologists [20]. The biggest initiative for proposing automated approaches in this direction was the Radiological Society of North America (RSNA) 2018 Bone Age Challenge [51]. This challenge provided a database of circa 12,000 radiographs of subjects from 0 to 19 years, labeled with the BA given by radiologists, following the GP method. Although the first places achieved MAEs of 4.26, 4.35, and 4.38 months, these results are not comparable with our results because the aim of our study was to estimate CA, and the RSNA challenge goal was to propose models for predicting the BA given by radiologists [51]. In addition, it is worth mentioning that however large the sample provided for the challenge, only 0.74% (94/12,612) of the sample consisted of 18- and 19-year-old subjects, which are important legal ages.

For studies that employ BA concepts to predict the CA of subjects, there are studies by Dallora et al [52] and Stern et al [53]. Both employ MRI as the medical imaging of choice, and most importantly, they are not based on traditional BAA to make their predictions of CA. They employ deep learning technology, which is able to learn the important features in the images and then perform regression or classification [54]. The reasoning behind using deep learning to interpret images and learn features is that it is difficult for humans to translate image features into descriptive means, and it is easy to lose information on the process. On the other hand, this problem has a reduced risk of occurring with algorithms able to analyze images pixel by pixel [55]. Dallora et al [52] used knee MRI images and achieved an MAE of 0.793 years for male subjects in the range of 14 to 20 years, and 0.988 years for female subjects in the range of 14 to 19 years. Stern et al [53] used MRI images of the hand and achieved an MAE of 0.82 years for male subjects in the range of 13 to 19 years. A previous study by Stern et al [56] proposed a deep learning multifactorial approach that used MRI volumes of the hand, clavicle, and teeth to estimate the CA of male subjects aged 13 to 25 years, achieving an MAE of 1.01 years. The study by Tang et al [57] used MRI for CA estimation in adolescents from 12 to 17 years, which leaves out the legal age of 18, using artificial neural networks. This was also a multifactorial approach that considered the subjects' height, weight, and bone marrow composition intensity quantified by MRI and TW3 assessment, achieving a mean disparity (comparison between the mean CA for all subjects and the mean estimated age for all subjects) of 0.1 years. This study also demonstrated that the BA given by the TW3 method was consistently lower than that of the subjects' CA. The study by Hillewig et al [58] investigated a multiple ROI approach that considered primarily the radiologists' assessment of MRI images of the clavicle, but also the assessment of x-rays of the hand and wrist area, with the aim of determining whether an individual is younger or older than 18 years, considering a sample of subjects from 16 to 26 years. It was evidenced that the clavicle assessment in stage IV (according to the Schmeling et al [9] and Kreitner et al [13] staging systems) was particularly important for age determination; however, in cases where staging is challenging for radiologists, the assessment of the hand and wrist area is essential.

### Limitations

Regarding limitations of the study, it could be argued that due to the high number of classes in the multiclass classification, the sample size in each class would not be large enough to build a generalizable model. However, to address this issue, we employed methods to ensure that the model would not overfit and for not choosing the most overly optimistic choice given by the nested cross-validation. In addition, during data collection, we ensured a uniform number of subjects in each class to guarantee a balanced data set.

The selected ROI for this work took into consideration the stress levels for the minors and young adult subjects with regard to the MRI examination. Hence, the clavicle and arm were not considered because it would require the subjects to go head in the MRI machine, which could cause discomfort and stress to the young subjects due to loud noises and small enclosed spaces. In addition, the clavicle has a high risk of producing moving artifacts due to breathing movements. On the practical side, the examination time was on average 15 min, and the inclusion of these 2 regions would take approximately double the time.

### Conclusions

This paper presented models for CA estimation and minors versus adults classification (on a threshold of 18 years) using ML algorithms. The models were trained with radiologists assessment of the calcaneus, distal tibia, proximal tibia, distal femur, and radius; and additional information regarding physical activity level, parents' origin, type of residence during upbringing, and a self-assessed Tanner Scale of pubertal growth. The models proposed for the classification of minor versus adults produced accuracies of 90% and 84% for male and female subjects, respectively, with very high recalls for the classification of minors. However, for the chronological age estimation for the 8 age groups, ranging from to 14 to 21, the variables in the model did not turn out to be precise enough for estimating the exact CA, only showing acceptable values of MAE for the ages of 14 and 15 years.

Future research should focus on applying deep learning technology for the estimation of CA using multiple ROIs.

## Appendix

Multimedia Appendix 1 Data characteristics of the variables collected through the questionnaire and BMI.

Multimedia Appendix 2 Results from the assessment of magnetic resonance images of male subjects.

Multimedia Appendix 3 Results from the assessment of magnetic resonance images of female subjects.

## Abbreviations

AUC: area under the curve
BA: bone age
BAA: bone age assessment
CA: chronological age
GP: Greulich-Pyle
KNN: K-nearest neighbors
MAE: mean absolute error
MLP: multilayer perceptron
MRI: magnetic resonance imaging
RMSE: root mean squared error
ROI: region of interest
RSNA: Radiological Society of North America
SLR: systematic literature review
SVM: support vector machines
TW: Tanner-Whitehouse
