# Supplementary material for: Chronological Age Assessment in Young Individuals Using Bone Age Assessment Staging and Nonradiological Aspects: Machine Learning Multifactorial Approach
Source: JMIR Med Inform. 2020 Sep 21;8(9):e18846. doi: 10.2196/18846 (PMC7536601; doi:10.2196/18846)
Supplement: Multimedia Appendix 1 [file medinform_v8i9e18846_app1.pdf]

Multimedia appendix 1 – Data characteristics of the variables collected through the questionnaire and BMI

Supplementary Table 1. Number of cases of the Residence variable discriminated by age on male subjects

**Residence – male subjects**				
		**Rented**	**Owned**	**Total**
Age	14	7	50	57
	15	8	48	56
	16	8	52	60
	17	7	51	58
	18	6	47	53
	19	9	49	58
	20	14	39	53
	21	6	53	59
Total		65	389	454

Supplementary Table 2. Number of cases of the Residence variable discriminated by age on female subjects

**Residence – female subjects**				
		**Rented**	**Owned**	**Total**
Age	14	8	51	59
	15	11	47	58
	16	6	50	56
	17	7	53	60
	18	8	50	58
	19	15	42	57
	20	11	45	56
	21	12	48	60
Total		78	386	464

Supplementary Table 3. Number of cases of the Physical Activity variable discriminated by age on male subjects

Physical Activity – male subjects						
		**Inactive**	**Little Active**	**Active**	**Highly active**	**Total**
Age	14	1	17	35	5	58
	15	1	15	30	8	54
	16	1	19	36	4	60
	17	4	16	32	6	58
	18	1	9	36	4	50
	19	3	15	36	3	57
	20	4	9	35	4	52
	21	6	14	34	3	57
Total		21	114	274	37	446

Supplementary Table 4. Number of cases of the Physical Activity variable discriminated by age on female subjects

Physical Activity – female subjects						
		**Inactive**	**Little Active**	**Active**	**Highly active**	**Total**
Age	14	6	15	28	9	58
	15	2	23	23	9	57
	16	6	17	32	2	57
	17	2	22	28	8	60
	18	2	15	38	3	58
	19	0	13	33	9	55
	20	3	17	28	9	57
	21	4	14	33	8	59
Total		25	136	243	57	461

Supplementary Table 5. Number of cases of the Parent Origin variable discriminated by age on male subjects

**Parent Origin – male subjects**					
		**no foreign-born parents**	**one foreign-born parent**	**both foreign-born parents**	**Total**
Age	14	39	15	4	58
	15	39	12	5	56
	16	39	15	6	60
	17	37	16	5	58
	18	31	17	5	53
	19	35	9	14	58
	20	35	10	8	53
	21	34	14	11	59
Total		289	108	58	455

Supplementary Table 6. Number of cases of the Parent Origin variable discriminated by age on female subjects

**Parent Origin – female subjects**					
		**no foreign-born parents**	**one foreign-born parent**	**both foreign-born parents**	**Total**
Age	14	38	17	2	57
	15	33	18	7	58
	16	45	8	4	57
	17	40	14	6	60
	18	32	15	12	59
	19	39	11	7	57
	20	35	11	10	56
	21	25	19	16	60
Total		287	113	64	464

Supplementary Table 7. Number of cases of the Tanner Scale variable discriminated by age on male subjects

Tanner scale – male subjects							
		**Stage 1**	**Stage 2**	**Stage 3**	**Stage 4**	**Stage 5**	**Total**
Age	14	1	6	19	30	2	58
	15	1	0	11	37	7	56
	16	0	0	3	39	17	59
	17	0	0	2	28	28	58
	18	0	0	1	18	34	53
	19	0	0	0	11	46	57
	20	0	0	1	7	44	52
	21	0	0	0	5	54	59
Total		2	6	37	175	232	452

Supplementary Table 8. Number of cases of the Tanner Scale variable discriminated by age on male subjects

Tanner scale – female subjects					
		**Stage 3**	**Stage 4**	**Stage 5**	**Total**
Age	14	31	27	1	59
	15	12	36	10	58
	16	8	39	10	57
	17	3	41	16	60
	18	1	32	25	58
	19	3	19	35	57
	20	2	12	43	57
	21	1	16	43	60
Total		61	222	183	466

Supplementary Table 9. Minimum, maximum and mean regarding the Body Mass Index (BMI) variable discriminated by age on male subjects

Body Mass Index (BMI) – male subjects					
		**Minimum**	**Maximum**	**Mean**	**Total**
Age	14	15.96	27.06	19.89	58
	15	15.63	27.00	20.66	56
	16	17.14	30.00	21.10	60
	17	16.60	31.57	21.43	58
	18	16.91	40.29	22.60	53
	19	16.66	37.87	22.60	58
	20	16.66	33.71	23.45	53
	21	16.33	35.43	22.66	59

Supplementary Table 10. Minimum, maximum and mean regarding the Body Mass Index (BMI) variable discriminated by age on female subjects

Body Mass Index (BMI) – female subjects					
		**Minimum**	**Maximum**	**Mean**	**Total**
Age	14	16.15	34.15	21.39	59
	15	17.72	28.93	21.97	58
	16	16.23	28.12	21.86	57
	17	13.52	34.91	22.74	60
	18	17.51	41.21	23.52	59
	19	16.65	36.00	21.98	57
	20	17.58	36.00	22.52	57
	21	17.15	29.05	22.82	60
