# Supplementary material for: Chronological Age Assessment in Young Individuals Using Bone Age Assessment Staging and Nonradiological Aspects: Machine Learning Multifactorial Approach
Source: JMIR Med Inform. 2020 Sep 21;8(9):e18846. doi: 10.2196/18846 (PMC7536601; doi:10.2196/18846)
Supplement: Multimedia Appendix 2 [file medinform_v8i9e18846_app2.pdf]

Supplementary Table 1. results from the assessment of MRI images of male subjects

		Calcaneus assessment results - male subjects								
		Age								Total
		14	15	16	17	18	19	20	21	
CALCANEUS	Stage3	1	1	0	0	0	0	0	0	2
	Stage4a	23	8	2	0	0	0	0	0	33
	Stage4b	7	6	2	0	0	0	0	0	15
	Stage4c	16	11	10	1	0	1	0	0	39
	Stage5	11	30	46	57	53	57	53	59	366
Total		58	56	60	58	53	58	53	59	455
		Distal Tibia assessment results - male subjects								
		Age								Total
		14	15	16	17	18	19	20	21	
DISTAL TIBIA	Stage4a	20	10	2	0	0	0	0	0	32
	Stage4b	18	12	4	0	0	0	0	0	34
	Stage4c	17	23	19	6	1	2	2	0	70
	Stage5	3	11	35	52	52	56	51	59	319
Total		58	56	60	58	53	58	53	59	455
		Proximal Tibia assessment results - male subjects								
		Age								Total
		14	15	16	17	18	19	20	21	
PROXIMAL TIBIA	Stage4a	25	16	6	0	0	0	0	0	47
	Stage4b	24	16	7	2	0	0	0	0	49
	Stage4c	9	23	35	23	5	2	0	0	97
	Stage5	0	1	12	33	48	56	53	59	262
Total		58	56	60	58	53	58	53	59	455
		Distal Femur assessment results - male subjects								
		Age								Total
		14	15	16	17	18	19	20	21	
DISTAL FEMUR	Stage4a	23	21	11	0	0	0	0	0	55
	Stage4b	25	23	15	7	2	0	0	0	72
	Stage4c	10	12	24	25	6	1	0	0	78
	Stage5	0	0	10	26	45	57	53	59	250
Total		58	56	60	58	53	58	53	59	455
		Radius assessment results - male subjects								
		Age								Total
		14	15	16	17	18	19	20	21	
RADIUS	Stage3	11	3	1	0	0	0	0	0	15
	Stage4a	38	33	22	11	0	1	0	0	105
	Stage4b	7	15	12	12	4	0	0	0	50
	Stage4c	2	4	20	20	17	5	1	0	69
	Stage5	0	1	5	15	32	52	52	59	216
Total		58	56	60	58	53	58	53	59	455
