# Supplementary material for: Chronological Age Assessment in Young Individuals Using Bone Age Assessment Staging and Nonradiological Aspects: Machine Learning Multifactorial Approach
Source: JMIR Med Inform. 2020 Sep 21;8(9):e18846. doi: 10.2196/18846 (PMC7536601; doi:10.2196/18846)
Supplement: Multimedia Appendix 3 [file medinform_v8i9e18846_app3.pdf]

Supplementary Table 2. results from the assessment of MRI images of female subjects

Calcaneus assessment results - female subjects										
		Age								Total
		14	15	16	17	18	19	20	21	
CALCANEUS	Stage4a	1	0	0	0	0	0	0	0	1
	Stage4b	3	0	0	0	0	0	0	0	3
	Stage4c	12	6	2	1	0	0	0	0	21
	Stage5	43	52	55	59	59	57	57	60	442
Total		59	58	57	60	59	57	57	60	467
Distal Tibia assessment results - female subjects										
		Age								Total
		14	15	16	17	18	19	20	21	
DISTAL TIBIA	Stage4a	2	0	0	0	0	0	0	0	2
	Stage4b	6	0	0	0	0	0	0	0	6
	Stage4c	21	9	4	1	0	0	0	0	35
	Stage5	30	49	53	59	59	57	57	60	424
Total		59	58	57	60	59	57	57	60	467
Proximal Tibia assessment results - female subjects										
		Age								Total
		14	15	16	17	18	19	20	21	
PROXIMAL TIBIA	Stage4a	10	3	0	0	0	0	0	0	13
	Stage4b	10	4	0	0	0	0	0	0	14
	Stage4c	28	22	7	2	0	0	0	0	59
	Stage5	11	29	50	58	59	57	57	60	381
Total		59	58	57	60	59	57	57	60	467
Distal Femur assessment results - female subjects										
		Age								Total
		14	15	16	17	18	19	20	21	
DISTALA FEMUR	Stage4a	19	7	0	0	0	0	0	0	26
	Stage4b	19	11	2	1	0	0	0	0	33
	Stage4c	17	18	16	8	1	0	0	0	60
	Stage5	4	22	39	51	58	57	57	60	348
Total		59	58	57	60	59	57	57	60	467
Radius assessment results - female subjects										
		Age								Total
		14	15	16	17	18	19	20	21	
RADIUS	Stage3	2	0	0	0	0	0	0	0	2
	Stage4a	26	13	3	1	0	0	0	0	43
	Stage4b	18	11	8	1	0	0	0	0	38
	Stage4c	11	26	22	13	6	0	0	0	78
	Stage5	2	8	24	45	53	57	57	60	306
Total		59	58	57	60	59	57	57	60	467
